# Dysphagia, Speech, Voice, and Trismus following Radiotherapy and/or Chemotherapy in Patients with Head and Neck Carcinoma: Review of the Literature

**DOI:** 10.1155/2016/6086894

**Published:** 2016-09-19

**Authors:** B. J. Heijnen, R. Speyer, B. Kertscher, R. Cordier, K. W. J. Koetsenruijter, K. Swan, H. Bogaardt

**Affiliations:** ^1^Department of Otorhinolaryngology and Head and Neck Surgery, Leiden University Medical Centre, Leiden, Netherlands; ^2^College of Healthcare Sciences, James Cook University, Townsville, QLD, Australia; ^3^Kantonsspital Winterthur, Winterthur, Switzerland; ^4^School of Occupational Therapy and Social Work, Curtin University, Perth, WA, Australia; ^5^Department of Family Medicine, School of Health Professions Education (SHE), Maastricht University Medical Centre, Maastricht, Netherlands; ^6^Speech Pathology Service, Gold Coast Health, Brisbane, QLD, Australia; ^7^Discipline of Speech Pathology, Faculty of Health Sciences, The University of Sydney, Sydney, NSW, Australia

## Abstract

*Introduction*. Patients with head and neck cancer suffer from various impairments due to the primary illness, as well as secondary consequences of the oncological treatment. This systematic review describes the effects of radiotherapy and/or chemotherapy on the functions of the upper aerodigestive tract in patients with head and neck cancer.* Methods*. A systematic literature search was performed by two independent reviewers using the electronic databases PubMed and Embase. All dates up to May 2016 were included.* Results*. Of the 947 abstracts, sixty articles met the inclusion criteria and described one or more aspects of the sequelae of radiotherapy and/or chemotherapy. Forty studies described swallowing-related problems, 24 described voice-related problems, seven described trismus, and 25 studies described general quality of life. Only 14 articles reported that speech pathologists conducted the interventions, of which only six articles described in detail what the interventions involved.* Conclusion*. In general, voice quality improved following intervention, whereas quality of life, dysphagia, and oral intake deteriorated during and after treatment. However, as a consequence of the diversity in treatment protocols and patient characteristics, the conclusions of most studies cannot be easily generalised. Further research on the effects of oncological interventions on the upper aerodigestive tract is needed.

## 1. Introduction

Head and neck oncological patients suffer from various functional, physical, and emotional impairments due to both the primary illness and the secondary consequences of the tumor treatment [1]. The oncological treatment of head and neck tumors depends on the location and the stage of the tumor, as well as the treatment preferences of the individual patient. Head and neck oncological treatment can include surgery, radiotherapy, chemotherapy, or combinations of these. The impact of head and neck oncological treatments on the anatomical structures, organ function, and the quality of life (QoL) should not be underestimated [2]. For instance, the implications of loss of function for people treated nonsurgically for head and neck cancer (HNC) and its detrimental effects on functioning and QoL are well documented [3].

In order to assist people with dysphagia to adjust to and live successfully with the sequelae of the primary condition, speech pathologists managing this caseload need to ensure posttreatment services are available [4] that address not only the physical but also the emotional and psychosocial needs. A qualitative study by Nund et al. [5] exploring dysphagia management by speech pathologists suggests that care givers generally feel ill-prepared for their role. Furthermore, this study suggests that clinicians should provide adequate and timely training and support to carers. Furthermore, Krisciunas et al. [6] concluded that within speech pathology there is no standardised therapy for HNC patients and scant evidence to support any particular protocol. As a result, institutions and individual speech pathologists need to develop their own protocols based on “standard” practices or anecdotal evidence.

Evidence-based practice (EBP) is hailed to be paramount in the practice of speech pathology [7]. The American Speech-Language-Hearing Association (ASHA) defines evidence-based practice as “…an approach in which current, high-quality research evidence is integrated with practitioner expertise and client preferences and values, into the process of making clinical decisions” [8]. Essentially, EBP involves moving the foundation for clinical decisions from clinical protocols centered solely on expert opinion to the integration of clinical expertise, the best current research evidence, and individual client values. To facilitate EBP in healthcare, clinical practice guidelines can be developed to summarise clinically relevant evidence [9].

Several reviews have been published about the outcomes after radiotherapy and/or chemotherapy in HNC patients (e.g., Frowen and Perry [10]; Jacobi et al. [11]; van der Molen et al. [12]; Paleri et al. [13]; Roe et al. [14]). Most of the reviews focused on selected functional domains in populations with HNC: health-related QoL [15], swallowing [13, 14, 16], and voice and speech [11]. Only the review by van der Molen et al. [12] covered a wider range of functional outcomes in patients with advanced HNC, including swallowing, mouth opening, nutrition, pain, and QoL. Further, the purpose of some studies was to provide evidence-based clinical guidelines (e.g., Paleri et al. [13]) and they did not perform systematic literature searches in line with the PRISMA guidelines [17]. As such, even though a number of reviews have been published over the last ten years, a comprehensive updated systematic review is needed that includes all functional domains affected by radiotherapy and/or chemotherapy in patients with head and neck carcinoma.

A systematic review was conducted to describe the effects of radiotherapy and/or chemotherapy on functions of the upper aerodigestive tract in patients with HNC and examined the evidence of interventions by speech pathologists.

## 2. Methods

A systematic literature search was performed by two independent reviewers. The electronic biomedical databases PubMed and Embase were used (search period from start of database until 5 May 2016). The searches were limited to English language publications. In PubMed the MeSH terms* larynx* or* hypopharynx* were combined with all MeSH terms related to head and neck neoplasms (Table 1). Next, the results were linked to all MeSH terms for chemotherapy or radiotherapy, after which the outcome was combined with all MeSH terms found for dysfunctions of the upper aerodigestive tract and limited with adults +19 years. The exact syntax of the literature search is presented in Table 1.

In Embase the thesaurus terms* larynx* or* hypopharynx* were linked to* neoplasm* and* radiotherapy* or* chemotherapy*. Next, the search outcome was combined with the following terms:* dysphagia*,* speech*,* speech disorder*,* voice*,* dysphonia*,* xerostomia*,* quality of life*,* dysarthria, or trismus* (see Table 1).

To identify the most recent publications, the search was complemented by free text words in PubMed and Embase (for the period after April 2015 until May 2016). Truncation symbols and wildcards were used to search for variant forms of words or word extensions:* Laryn* ^*∗*^,* pharyn* ^*∗*^ or* hypopharyn* ^*∗*^ were combined with* cancer* ^*∗*^,* neoplasm* ^*∗*^,* tumour* ^*∗*^ or* carcinoma* ^*∗*^. Furthermore, these free text words were combined with* radiation* ^*∗*^,* radiotherap* ^*∗*^,* chemotherap* ^*∗*^,* adjuvant therap* ^*∗*^ or* radiochemotherap* ^*∗*^ and, finally, combined with* deglut* ^*∗*^,* swallow* ^*∗*^,* dysphag* ^*∗*^,* speech* ^*∗*^,* voic* ^*∗*^,* articulat* ^*∗*^,* dysphon* ^*∗*^,* quality of life* ^*∗*^,* xerostom* ^*∗*^,* dysarthr* ^*∗*^ or* anarthr* ^*∗*^.

Only articles presenting both pre- and postintervention data of the upper aerodigestive tract functions of the participants were included. Review articles and studies with a population sample of less than 20 patients were excluded, as well as experiments on animals or articles not published in English. Furthermore, studies published before 1990, case reports, expert opinions, and articles describing combinations of therapy including surgical interventions were excluded.

Final decisions on inclusion were made based on the original articles by consensus between two expert reviewers in accordance with the PRISMA statement [17]. The reference lists of all the included articles were searched for additional literature. Next, the standard quality assessment QualSyst as described by Kmet et al. [18] was performed in order to evaluate the methodological strength and weaknesses of the included studies. All ratings were performed by two independent reviewers. After consensus, studies with poor methodology scores (<50%) were excluded. All included articles were classified according to the Australian National Health and Medical Research Council (NHMRC) Evidence Hierarchy [19]. Data were retrieved from all studies and tabulated; further details on selected speech pathology interventions were summarised separately.

## 3. Results

**Figure 1:**
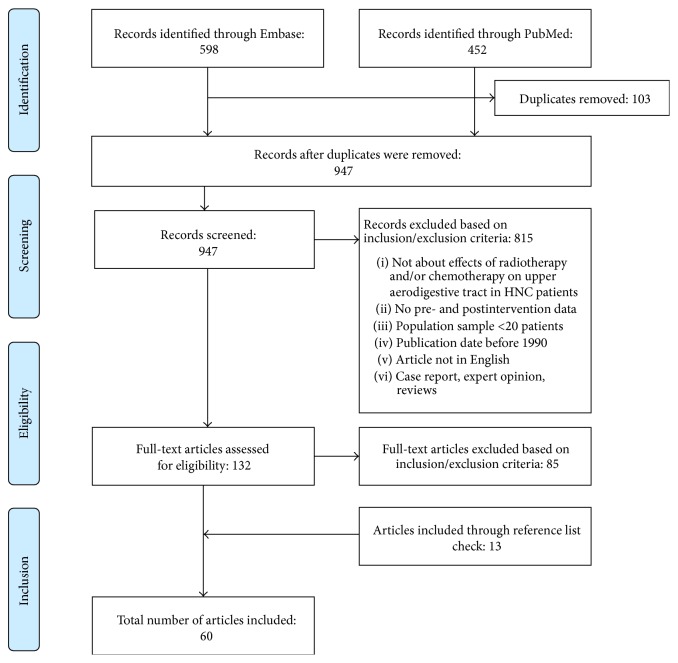
PRISMA flowchart.

Using MeSH or thesaurus terms, 304 articles were located in PubMed and 201 in Embase. Free text word searches resulted in another 148 articles in PubMed and 397 in Embase. The combination of these searches, without overlap, yielded 947 articles.  Figure 1 outlines the PRISMA reviewing process according to Moher et al. [20]. Sixty articles met all inclusion criteria.

Table 2 shows the outcomes of the QualSyst critical appraisal tool by Kmet et al. [18]. As all studies had sufficient methodological quality, no further studies were excluded; the overall methodological quality ranged from adequate to good with 0 studies ranked as poor, 3 studies as adequate, 3 studies as good, and 54 studies as strong. Based on the NHMRC Evidence Hierarchy [19], 6 studies were classified as level II evidence and 54 studies as level III evidence.

All 60 studies focused on different functions of the upper aerodigestive tract following radiotherapy and/or chemotherapy for HNC. The following constructs were evaluated across the different studies: communication (voice and speech), functions of the digestive tract (oral intake, weight loss, dysphagia, trismus, xerostomia, and tube dependency), QoL, and overall survival rates.

Table 3 provides a summary of the 60 retrieved observational and intervention studies that met the inclusion criteria. The first column presents the reference of the author(s). The second column represents the number of subjects, the third column represents the etiology of the head and neck malignancies, and the 4th column displays the staging of the malignancies. The 5th column shows whether voice and/or speech, digestive tract, and QoL were studied. The 6th and 7th columns show the evaluation techniques and the treatment, respectively. The 8th column presents the follow-up and the last column describes the author's key findings.

### 3.1. Voice and/or Speech Function

Twenty-four studies evaluated voice and/or speech function [21–43] with a follow-up time ranging from 1-month follow-up [42] to ten-year follow-up [43]. Most studies included patients with laryngeal tumors only; however 11 studies [22, 27, 29, 30, 33–37, 44] also included nonlaryngeal tumors. Seventeen studies [21, 23, 25, 26, 28, 30–32, 34–36, 38–43] included patients with low-grade tumors (i.e., T1 and T2) and 15 studies included patient with advanced tumors [22, 24, 27, 29–37, 40, 42, 44].

Nine studies [23, 28, 30, 32, 33, 35, 38, 42, 44] used acoustic analysis to evaluate voice quality, six studies [24–26, 34, 35, 39] used the Voice Handicap Index, and three studies [21, 38, 43] used videolaryngostroboscopy. In several studies, either descriptions of how voice quality was evaluated were missing or nonvalidated tools were used (e.g., patients self-reporting or trial-specific questionnaires). Only four studies [23, 31, 40, 44] reported whether the patient received any voice therapy.

All the studies reported good to excellent outcomes for voice quality at long-term follow-up. Some studies specifically reported pre- to posttreatment improvements of voice or speech quality following radiotherapy and/or chemotherapy [22, 23, 28]. However, other studies [36, 40, 42, 44] reported a deterioration after therapy at long-term follow-up. Al-Mamgani et al. [26] found a better voice outcome in case of single vocal cord irradiation compared with irradiation of the whole larynx. Mittal et al. [37] concluded that radiation with tissue/dose compensation (TDC) improved articulatory outcome compared to radiation without TDC.

### 3.2. Functions of the Digestive Tract

Forty studies [16, 22, 29, 34–37, 42, 45–76] describe the effects of radiotherapy and/or chemotherapy on the functions of the digestive tract and used a variety of outcome measures. Of these 40 studies, 16 studies [16, 35, 37, 45, 49, 53, 54, 56, 58, 63, 65, 67, 69, 70, 73, 74] used videofluoroscopy to measure physiological changes in swallowing function. Eight studies [29, 35, 46, 47, 49, 55, 60, 75] used feeding tube dependency as a (dichotomous) outcome, whereas seven studies [35, 46, 57, 65, 66, 69, 73] described the level of oral intake in more detail. Only four studies [35, 36, 56, 76] used a condition specific validated measure for swallowing disorders (e.g., MDADI).

With regard to nutritional status, five studies [29, 60, 62, 63, 73] used the body mass index as an outcome or reported specifically on weight gain or loss. Seven studies [35, 36, 48, 52, 64, 73, 74] used the presence of trismus as an outcome by reporting on the maximum distance of mouth opening. Saliva flow (as a measure of xerostomia) was used in four studies [36, 37, 68, 71].

Follow-up times in these studies range from immediately after therapy [62] to 6 years after therapy [35], describing both low stage tumors and more advanced tumors. Thirty-two studies used the TNM-classification system, stage was described in six other studies, and the remaining two studies did not report on tumor stage or grade. However, it was unclear whether the clinical TNM-score or the pathological TNM-score was used to describe the severity of the disease. Eight studies [16, 35, 46, 48, 52, 56, 57, 69, 73, 74] described whether the patients received functional treatment (by a speech pathologist); the remainder of the articles did not mention whether the patient received any additional treatment.

Nine studies reported impaired swallowing function following radiotherapy and/or chemotherapy [36, 45, 50, 54–56, 69, 75, 76].

Five studies [16, 51, 59, 61, 66] showed that swallowing was least affected at baseline, worst immediately following posttreatment (0–3 months after treatment), and improved by 6–12 months after treatment and later. However, swallowing usually did not return to pretreatment functioning level. In four studies [49, 53, 58, 74], a relation between dose-volume, dysphagia, and aspiration was found. Caudell et al. [49] showed that a mean radiation dosage >41 Gy with >24% volume of the larynx being radiated was associated with increased percutaneous endoscopic gastrostomy (PEG) dependency and aspiration. Akst et al. [46] correlated advanced tumor stage and age >60 years with a deterioration of swallowing.

Ackerstaff et al. [22] demonstrated improved oral intake postradiotherapy and/or chemotherapy. Stenson et al. [70] stated that weight remained unchanged after treatment (via oral route), whereas Nourissat et al. [62] described a mean weight loss of 2.2 kg posttreatment.

### 3.3. QoL

Twenty-five studies [22, 24, 25, 27–29, 31, 32, 35, 36, 42, 44, 47, 51, 53, 55, 59, 62, 64, 68, 72, 76–79] described the short- and long-term effects of treatment for HNC on patients' general QoL. The European Organization for Research and Treatment of Cancer (EORTC) C30-questionnaire was used in fifteen studies [22, 24, 25, 27, 31, 32, 35, 42, 47, 55, 62, 64, 77–79] and the more HNC specific EORTC H&N35 was used in thirteen studies [22, 24, 25, 31, 32, 35, 36, 42, 47, 64, 77–79]. Other questionnaires that were used included the University of Washington QoL Questionnaire (UWQoL) [53, 68, 72, 76], the Head and Neck QoL or HNQoL [53, 72], and the Xerostomia related QoL or XQoL [68, 72]. Follow-up time for QoL was up to six years after treatment [35], including patients with tumors that were early staged and patients with advanced tumors.

Although three studies [28, 72, 77] demonstrated improvements in QoL, four studies [22, 36, 42, 54] reported a decrease in general QoL as a result of radiotherapy and/or chemotherapy. Bansal et al. [27] found a significant decline in physical, social, and emotional functioning as well as in global health scores following a course of radiotherapy. However, the patients' functional scores improved one month after treatment but did not reach pretreatment levels. The health-related QoL (HRQoL) scores of the majority of patients in the Bottomley et al. [78] study returned to baseline at 48-month follow-up. These findings support the findings of Ackerstaff et al. [22], Cohen et al. [51], Karlsson et al. [32], List et al. [59], and Wilson et al. [76], who suggested that HRQoL deteriorates significantly immediately after treatment, with variable degrees of improvement 3–72 months after treatment.

### 3.4. Reported (Efficacy of) Speech Pathology Interventions

We assessed the speech pathology interventions against the following criteria: (a) whether a detailed description of the intervention was provided; (b) whether the authors provided a description of treatment duration and intensity; and (c) what the speech pathology intervention outcomes were. The reported efficacy of 14 speech pathology intervention studies aimed at addressing problems in dysphagia, speech, voice, and trismus is summarised in Table 4.

Of the 60 articles included in this review, 14 studies [16, 23, 29, 31, 35, 40, 44, 46, 48, 56, 57, 69, 73, 74] reported whether there was any treatment for the sequelae of radiotherapy and/or chemotherapy. Of these intervention studies, five focused on voice-related problems [23, 31, 35, 40, 44], two focused on trismus [48, 52], seven focused on swallowing disorders [16, 35, 46, 56, 57, 69, 73], and one study reported on both swallowing disorders and trismus [74].

The three studies that investigated the treatment of trismus [48, 52, 74] presented the most detailed information on what the interventions involved. The study by Dijkstra et al. [52] described a wide variety of trismus-specific therapies, suggesting that most patients received a combination of therapies. The patients in the van der Molen et al. [44] and Kraaijenga et al. [35] studies did not receive any speech therapy. The remainder of the studies reported that patients received speech therapy; however, most of these studies did not provide specific data on treatment duration or intensity. None of the voice-related studies provided information on the specific exercises prescribed to patients except Karlsson et al. [31].

Of the eight studies on swallowing disorders, only Kotz et al. [57] and van der Molen et al. [73] described the prescribed exercises in detail. The aim of the latter study was to compare the effectiveness of experimental rehabilitation to standard rehabilitation in 49 advanced HNC patients. The authors concluded that preventive rehabilitation is feasible and effective in reducing the extent and/or severity of various functional short-term effects of chemoradiotherapy [74]. This finding is supported by the 6-year follow-up study by Kraaijenga et al. [35]. Kotz et al. [57] described a temporary improvement. These are the only studies that provided detailed information about the speech pathology intervention and reported on the effectiveness of the intervention.

## 4. Discussion

In total, 60 studies met the inclusion criteria. The studies described the effects of radiotherapy and/or chemotherapy on the functions of the upper aerodigestive tract in patients with HNC. The articles yielded by this systematic review vary in their findings regarding tumor characteristics and treatment modalities. As a result of this variability, no statistical pooling was possible. We also set out to investigate the involvement of speech pathologists in treating patients with HNC.

When considering treatment outcomes, voice quality worsened at the start of radiotherapy and/or chemotherapy but eventually improved after therapy finished. Dysphagia can be a major side effect of HNC and its treatment. The high incidence of dysphagia in this study population can cause serious secondary consequences, such as malnutrition, dehydration, an increased risk of aspiration, and, at worst, death [80]. As dysphagia is a common sequela to oncological treatment, early detection and treatment are needed to avoid or minimise serious secondary complications [81].

The general description of the study population in Table 3 shows that there was great variability in both the location of the tumor and the grading/staging, making comparisons of these studies difficult. As the follow-up times varied in each study, the outcomes may be noncomparable. Thus, this review shows that there is a need for more standardised approaches to research in this field.

Additionally, a large range of outcome measures were used, some of which are not validated. This calls into question the reliability of results reported in some of the studies. The use of validated and standardised assessments in future research would provide more robust findings.

When considering the functional outcomes of radiotherapy and/or chemotherapy, one of the most important factors is whether the patient had received voice or swallowing therapy. Interestingly, only 14 of the 60 included studies reported whether the patients received any speech therapy. Thus, in 46 articles functional results, such as voice quality, are presented with no specification of whether the patient received therapy. As some of these studies have a follow-up of >2 years, it is fair to assume that patients sought help for voice or swallowing problems. Therefore, the involvement of speech therapy may be underreported, suggesting that the presented outcomes in these studies are biased and raise questions about their reliability.

When information was provided about treatment, only six articles [31, 48, 52, 57, 73, 74] described in detail the treatment intensity as the actual treatment. Furthermore, these studies are the only five that include conclusions about the efficacy of speech therapy in this specific population. In the context of EBP, this finding demonstrates the need for more research into the efficacy of speech pathology interventions for patients with HNC receiving radiotherapy and/or chemotherapy.

To enable the objective reporting of the effectiveness of radiation and/or chemotherapy, baseline measurements of different aspects of voice quality and swallowing are required. To manage expectations, healthcare professionals and patients need to be made aware that some aspects of both voice and swallowing commonly do not recover to the level prior to the oncological intervention [16]. Regarding effectiveness of voice treatments, the following multidimensional assessment is recommended [82]: a videolaryngostroboscopy recording of the laryngeal structures and the vocal fold vibration; an acoustic and a perceptual analysis of voice; a voice-related questionnaire on QoL (e.g., the Voice Handicap Index) [83]; and a functional health status questionnaire. Such a protocol would be in line with the recommendations for functional assessment of voice pathology as described by the Committee on Phoniatrics of the European Laryngological Society [84].

When describing aspects of swallowing function, both fiber optic endoscopic evaluation of swallowing and videofluoroscopy are considered to be the gold standard in dysphagia assessment [85]. In addition to these tools, questionnaires on HRQoL and functional health status are recommended and should be integrated in the overall swallowing assessment protocol. Repeated measurements of outcome measures should be performed in order to monitor any side effects of the oncological intervention, to detect spontaneous recovery, and to measure the effects of the speech pathology interventions. Apart from baseline measurements, posttreatment and follow-up measurements should be used to monitor functional and QoL outcomes.

Additional research is needed to develop clinical practice guidelines to support evidence-based practice in the area of dysphagia, speech, voice, and trismus following radiotherapy and/or chemotherapy in patients with head and neck carcinoma. These practice guidelines should bring together the best available current evidence within a specific clinical area, formulating evidence-based recommendations for clinicians and present choices between different interventions that have an impact on health and use of resources [86]. This systematic review summarised the effects of radiotherapy and/or chemotherapy on the function of the upper aerodigestive tract in patients with head and neck cancer. However, because of the marked variation in treatment protocols and patient characteristics, outcome data from the included studies cannot be easily generalised. Recommendations for future studies advocate the use of a multidimensional assessment protocol, using well-validated measures and standardised pretreatment, posttreatment, and follow-up measurements, thus allowing for future meta-analysis of homogeneous outcomes.

## 5. Conclusion

The studies included in this systematic review described a wide variety of outcomes in patients with HNC following radiotherapy and/or chemotherapy. The findings about the long-term functional implications of radiotherapy and/or chemotherapy in patients with HNC are inconclusive as a result of the wide range of outcome measures used and the possible influence of underreported speech therapy.

Future researchers need to consider targeting more homogeneous groups using standardised treatment protocols to improve the treatment outcomes, thereby decreasing the side effects of the oncological treatments. Findings of these studies need to inform the decision-making process in the treatment of HNC so complications can be better predicted with due consideration of the possible negative side effects to the upper aerodigestive tract. Although the main objective of most studies was to determine curing rates, the importance of the functional implications of the side effects of oncology treatments should not be overlooked, particularly their impact on QoL. Finally, more research is needed to gain a full understanding of the complexity and variety in the effects of radiotherapy and/or chemotherapy on the functions of the upper aerodigestive tract following HNC.

## Figures and Tables

**Table 1:** Search strategies per literature database.

	Database and search terms	Limits	Number of records
Subject headings	*Embase*: (larynx/OR pharynx/OR hypopharynx/) AND (neoplasm/OR larynx disorder/OR pharynx disorder/OR larynx cancer/OR larynx carcinoma/OR pharynx cancer/OR pharynx carcinoma/) AND (radiotherapy/OR chemotherapy/OR chemoradiotherapy/OR adjuvant therapy/OR drug therapy/) AND (speech sound disorder OR speech/or speech disorder/OR swallowing/OR dysphagia/OR dysphonia/OR voice disorder/OR aphonia/OR speech intelligibility/OR xerostomia/OR dysarthria/OR esophagus speech/OR larynx prosthesis/OR trismus/OR “quality of life”/)	English	201

Subject headings	*PubMed*: (“Larynx”[Mesh] OR “Pharynx”[Mesh] OR “Hypopharynx”[Mesh]) AND (“Neoplasms”[Mesh] OR “Head and Neck Neoplasms”[Mesh] OR “Neoplasms, Second Primary”[Mesh] OR “Pharyngeal Neoplasms”[Mesh] OR “Oropharyngeal Neoplasms”[Mesh] OR “Tonsillar Neoplasms”[Mesh] OR “Nasopharyngeal Neoplasms”[Mesh] OR “Mouth Neoplasms”[Mesh] OR “Laryngeal Neoplasms”[Mesh] OR “Tongue Neoplasms”[Mesh] OR “Thyroid Neoplasms”[Mesh] OR “Salivary Gland Neoplasms”[Mesh] OR “Jaw Neoplasms”[Mesh] OR “Lip Neoplasms”[Mesh] OR “Thyroid Carcinoma, Anaplastic”[Mesh] OR “Neoplasms, Squamous Cell”[Mesh] OR “Neoplasms, Basal Cell”[Mesh] OR “Otorhinolaryngologic Neoplasms”[Mesh] OR “Hypopharyngeal Neoplasms”[Mesh] OR “Laryngeal Diseases”[Mesh] OR “Pharyngeal Diseases”[Mesh]) AND (“Radiotherapy”[Mesh] OR “Radiotherapy, Adjuvant”[Mesh] OR “Radiotherapy, High-Energy”[Mesh] OR “Radiotherapy, Image-Guided”[Mesh] OR “Radiotherapy, Intensity-Modulated”[Mesh] OR “Radiotherapy, Conformal”[Mesh] OR “Radiotherapy, Computer-Assisted”[Mesh] OR “Radiotherapy Planning, Computer-Assisted”[Mesh] OR “Radiotherapy Dosage”[Mesh] OR “Brachytherapy”[Mesh] OR “Radiosurgery”[Mesh] OR “Radiation Oncology”[Mesh] OR “Dose-Response Relationship, Radiation”[Mesh] OR “Consolidation Chemotherapy”[Mesh] OR “Induction Chemotherapy”[Mesh] OR “Maintenance Chemotherapy”[Mesh] OR “Chemotherapy, Adjuvant”[Mesh] OR “Chemotherapy, Cancer, Regional Perfusion”[Mesh] OR “Drug Therapy”[Mesh] OR “Drug Therapy, Combination”[Mesh] OR “Radiotherapy”[Mesh] OR “Radiation Dosage”[Mesh]) AND (“Articulation Disorders”[Mesh] OR “Speech”[Mesh] OR “Speech Sound Disorder”[Mesh] OR “Speech, Esophageal”[Mesh] OR “Speech, Alaryngeal”[Mesh] OR “Speech Intelligibility”[Mesh] OR “Speech Disorders”[Mesh] OR “Deglutition Disorders”[Mesh] OR “Deglutition”[Mesh] OR “Dysphonia” [Mesh] or “Voice Disorders” [Mesh] or “Hoarseness” [Mesh] or “Aphonia” [Mesh] OR “Xerostomia”[Mesh] OR “Dysarthria”[Mesh] OR “Larynx, Artificial”[Mesh] OR “Speech, Esophageal”[Mesh] OR “Trismus”[Mesh] OR “Quality of Life”[Mesh])	Adult: 19+ years English	304

Free text	*Embase*: (larynx^*∗*^ or pharynx^*∗*^ OR hypopharyn^*∗*^ OR laryngo^*∗*^ OR larynge^*∗*^) AND (cancer OR cancers OR neoplasm^*∗*^ OR tumour^*∗*^ OR tumor OR tumors OR carcinoma^*∗*^) AND (radiation^*∗*^ OR radiotherap^*∗*^ OR chemotherap^*∗*^ OR adjuvant therap^*∗*^ OR radiochemotherap^*∗*^) AND (deglut^*∗*^ OR swallow^*∗*^ OR dysphag^*∗*^ OR speech^*∗*^ OR voic^*∗*^ OR hoarse^*∗*^ OR aphon^*∗*^ OR rough^*∗*^ OR articulat^*∗*^ OR dysphon^*∗*^ OR (quality AND life) OR xerostom^*∗*^ OR dysarthr^*∗*^ OR anarthr^*∗*^ OR trismus)	Publication date: last year	397

Free text	*PubMed*: *As per Embase Free Text*	Publication date: from 2015/05/05 to 2016/05/05	148

**Table 2:** Methodological quality based on QualSyst critical appraisal tool by Kmet et al. 2004 [18] and NHMRC 1999 [19] evidence level of included articles.

Reference	Kmet score (%)	Methodological quality^1^	NHMRC level of evidence^2^
Aaltonen et al. 2014 [21]	25/28 (89%)	Strong	II
Ackerstaff et al. 2009 [22]	22/28 (79%)	Good	II
Agarwal et al. 2009 [23]	19/24 (79%)	Good	III-2
Agarwal et al. 2011 [45]	17/20 (85%)	Strong	III-3
Akst et al. 2004 [46]	17/20 (85%)	Strong	III-3
Al-Mamgani et al. 2012 [24]	19/20 (95%)	Strong	III-3
Al-Mamgani et al. 2012 [47]	21/22 (95%)	Strong	III-3
Al-Mamgani et al. 2013 [25]	21/22 (95%)	Strong	III-3
Al-Mamgani et al. 2015 [26]	21/22 (95%)	Strong	III-3
Bansal et al. 2004 [27]	14/24 (58%)	Adequate	III-3
Bibby et al. 2008 [28]	18/22 (82%)	Strong	III-2
Bottomley et al. 2014 [78]	24/28 (86%)	Strong	II
Buchbinder et al. 1993 [48]	14/26 (54%)	Adequate	III-1
Caudell et al. 2010 [49]	21/22 (95%)	Strong	III-3
Christianen et al. 2015 [50]	21/22 (95%)	Strong	III-3
Cohen et al. 2006 [51]	19/20 (95%)	Strong	III-3
Dornfeld et al. 2007 [29]	17/22 (77%)	Strong	III-3
Dijkstra et al. 2007 [52]	19/22 (86%)	Strong	III-3
Feng et al. 2007 [53]	19/22 (86%)	Strong	III-3
Feng et al. 2010 [54]	20/20 (100%)	Strong	III-3
Frowen et al. 2010 [16]	22/22 (100%)	Strong	III-2
Haderlein et al. 2014 [55]	17/20 (85%)	Strong	III-3
Hutcheson et al. 2014 [56]	18/20 (90%)	Strong	III-3
Jacobi et al. 2016 [30]	17/18 (94%)	Strong	III-3
Karlsson et al. 2015 [31]	26/28 (93%)	Strong	II
Karlsson et al. 2016 [32]	18/20 (90%)	Strong	III-3
Kazi et al. 2008 [33]	17/20 (85%)	Strong	III-2
Kerr et al. 2015 [34]	19/20 (95%)	Strong	III-2
Kotz et al. 2012 [57]	24/28 (86%)	Strong	II
Kraaijenga et al. 2014 [35]	19/20 (95%)	Strong	III-3
Kumar et al. 2014 [58]	19/20 (95%)	Strong	III-2
Lazarus et al. 2014 [36]	19/20 (95%)	Strong	III-3
List et al. 1999 [59]	15/18 (83%)	Strong	III-3
McLaughlin et al. 2010 [60]	19/20 (95%)	Strong	III-3
Mittal et al. 2001 [37]	16/20 (80%)	Strong	III-3
Murry et al. 1998 [61]	11/20 (55%)	Adequate	III-3
Niedzielska et al. 2010 [38]	17/20 (85%)	Strong	III-2
Nourissat et al. 2010 [62]	23/26 (88%)	Strong	III-3
Ottoson et al. 2014 [63]	19/22 (86%)	Strong	III-3
Pauli et al. 2013 [64]	19/22 (86%)	Strong	III-3
Pauloski et al. 2006 [65]	18/20 (90%)	Strong	III-3
Rademaker et al. 2003 [66]	17/20 (85%)	Strong	III-3
Remmelts et al. 2013 [39]	18/20 (90%)	Strong	III-3
Salama et al. 2008 [67]	17/20 (85%)	Strong	III-3
Sanguineti et al. 2014 [40]	19/20 (95%)	Strong	III-3
Scrimger et al. 2007 [68]	18/20 (90%)	Strong	III-3
Spector et al. 1999 [41]	17/22 (77%)	Good	III-3
Starmer et al. 2014 [69]	18/20 (90%)	Strong	III-3
Stenson et al. 2010 [70]	16/20 (80%)	Strong	III-3
Strigari et al. 2010 [71]	17/20 (85%)	Strong	III-3
Tuomi et al. 2015 [42]	18/20 (90%)	Strong	III-2
Urdaniz et al. 2005 [77]	18/20 (90%)	Strong	III-2
Vainshtein et al. 2015 [72]	20/24 (83%)	Strong	III-3
van der Molen et al. 2011 [73]	24/26 (92%)	Strong	II
van der Molen et al. 2012 [44]	16/20 (80%)	Strong	III-3
van der Molen et al. 2013 [74]	19/20 (95%)	Strong	III-3
Verdonck-de Leeuw et al. 1999 [43]	18/20 (90%)	Strong	III-2
Verdonck-de Leeuw et al. 2014 [79]	16/20 (80%)	Strong	III-2
Vlacich et al. 2014 [75]	18/20 (90%)	Strong	III-3
Wilson et al. 2011 [76]	18/20 (90%)	Strong	III-3

^1^Methodological quality: strong > 80%; good 60–79%; adequate 50–59%; poor < 50%.

^2^NHMRC evidence hierarchy designates the following hierarchy: level I (evidence obtained from a systematic review of all relevant RCTs), level II (evidence obtained from at least one properly designed RCT), level III-1 (evidence obtained from well-designed pseudo-RCTs [alternate allocation or some other method]), level III-2 (evidence obtained from comparative studies with concurrent controls and allocation not randomised [cohort studies], case control studies, or interrupted time series with a control group), level III-3 (evidence obtained from comparative studies with historical control, two or more single-arm studies, or interrupted time series without a parallel control group), and level IV (evidence obtained from case series, either posttest or pretest and posttest).

**Table 3:** Overview of included observational and intervention studies (*N* = 60) that met eligibility criteria.

Reference	Subjects	Carcinoma	Staging	Topic	Evaluation technique	Treatment(s)	Follow-up	Key findings/author's conclusions
Aaltonen et al. 2014 [21]	*N* = 56	Glottic = 56 (100%)	T1a = 56 (100%)	V	Videolaryngostroboscopy Expert rating (GRBAS) Patient self-rating (VAS) hoarseness and impact on everyday life	Group 1: laser surgery (*n* = 31) Group 2: RT (*n* = 25)	6, 24 months	Similar overall voice quality for both groups. Laser surgery yielded more breathiness compared to RT

Ackerstaff et al. 2009 [22]	*N* = 207	Oral cavity = 40 (19%) Oropharyngeal = 129 (62%) Hypopharyngeal = 38 (19%)	T3 = 65 (31%) T4 = 142 (69%)	V D QoL	EORTC QLQ-C30 EORTC QLQ-H&N35 Trial-specific questionnaires	Group 1: intra-arterial cisplatin 4 weekly (*n* = 104) + RT Group 2: intravenous cisplatin 3 weekly (*n* = 103) + RT	7 weeks; 3 months; 1, 2, 5 years	Both groups showed improved oral intake and voice quality, at 1-year follow-up often better compared to baseline

Agarwal et al. 2009 [23]	*N* = 50	Glottic = 50 (100%)	T1 = 33 (66%) T2 = 17 (34%)	V	Voice analysis Acoustic parameters: frequency, intensity, perturbation Patient-reported improvement in voice quality	RT	3–6 months	A trend for improvement in voice quality following RT was found

Agarwal et al. 2011 [45]	*N* = 47	Oropharyngeal Hypopharyngeal Laryngeal (No details provided)	T1 T2 T3 T4 (No details provided)	D	Videofluoroscopy PSS-HN	CRT	2, 6, 12 months	Significant impairment of swallowing was found: most frequently residue and aspiration

Akst et al. 2004 [46]	*N* = 196	Oral cavity = 12 (6%) Base of tongue = 41 (21%) Tonsil = 41 (21%) Other oropharyngeal = 15 (8%) Hypopharyngeal = 34 (17%) Laryngeal = 50 (26%) Unknown = 3 (1%)	T1 = 15 (8%) T2 = 42 (21%) T3 = 65 (33%) T4 = 70 (36%) Unknown = 4 (2%)	D	Presence of feeding tube Presence of tracheotomy Level of oral diet	CRT	3, 6, 12, 24 months	A majority of patients did not need a tracheotomy but need a feeding tube during treatment. At 1-year follow-up most patients had a (nearly) normal oral intake. Patients with tumor stage IV and age ≥ 60 had prolonged feeding tube use and slower recovery

Al-Mamgani et al. 2012 [24]	*N* = 170	Supraglottic = 121 (71%) Glottic = 49 (29%)	T3 = 170 (100%)	V QoL	EORTC QLQ-C30 EORTC QLQ-H&N35 VHI	Group 1: CRT (*n* = 48) Group 2: RT (*n* = 122)	2, 4, 6 weeks; 3, 6, 12 months	Adding chemotherapy to RT did not diminish QoL or voice handicap

Al-Mamgani et al. 2012 [47]	*N* = 176	Hypopharyngeal = 176 (100%)	T1 = 18 (10%) T2 = 55 (31%) T3 = 56 (32%) T4a = 35 (20%) T4b = 12 (7%)	D QoL	Tube dependency EORTC QLQ-C30 EORTC QLQ-H&N35	Group 1: CRT (*n* = 102) Group 2: RT (*n* = 74)	2, 4, 6 weeks; 3, 6 months; 1, 2 years	CRT significantly improved functional outcome. Acute toxicity increased but late radiation side effects did not increase

Al-Mamgani et al. 2013 [25]	*N* = 1050	Glottic = 1050 (100%)	T1a = 551 (52%) T1b = 168 (16%) T2a = 209 (20%) T2b = 122 (12%)	V QoL	EORTC QLQ-C30 EORTC QLQ-H&N35 VHI	RT	4, 6 weeks; 3, 6, 12, 18, 24, 36, 48 months	Excellent outcome with good QoL and VHI scores

Al-Mamgani et al. 2015 [26]	*N* = 30	Glottic = 30 (100%)	T1a = 30 (100%)	V	Laryngoscopy VHI	Single vocal cord RT	4, 6, 12 weeks; 6, 12, 18 months	Single vocal cord RT showed better voice quality compared to whole larynx RT

Bansal et al. 2004 [27]	*N* = 45	Base of tongue = 20 (44%) Tonsil = 10 (22%) Unknown = 15 (33%)	Stage III = 17 (38%) Stage IV = 23 (51%) Not reported = 5 (11%)	V QoL	Acute and late morbidity scoring of skin, oropharyngeal mucosa, salivary glands, larynx, and oesophagus (LENT/SOMA) EORTC QLQ-C30	RT	1, 4 months	During RT a decline in all QoL domains was found. QoL improved after 1 month but did not reach pre-RT levels

Bibby et al. 2008 [28]	*N* = 30	Glottic = 30 (100%)	T1 = 21 (70%) T2 = 9 (30%)	V QoL	Voice analysis Patient self-rating voice quality VR-QoL	RT	3, 6, 12 months	After RT expert-rated perceptual auditory outcomes, patient self-rated VAS and all subscales of VR-QoL showed significant improvement

Bottomley et al. 2014 [78]	*N* = 450	Laryngeal Hypopharyngeal (No details provided)	T2 T3 T4 (No details provided)	QoL	EORTC QLQ-C30 EORTC QLQ-H&N35	Group 1: sequential CRT (*n* = 224) Group 2: alternating CRT (*n* = 226)	6, 12, 18, 24, 36, 48 months	The HRQoL scores of the majority of patients returned to baseline after therapy. No group differences were found

Buchbinder et al. 1993 [48]	*N* = 21	No details provided	No details provided	D	MIO	Group 1: RT + unassisted exercise Group 2: RT + stacked tongue depressors combined + unassisted exercise Group 3: RT + TheraBite® system combined + unassisted exercise	2, 4, 6, 8, 10 weeks	The highest increase in MIO was reached in group 3

Caudell et al. 2010 [49]	*N* = 83	Nasal cavity = 3 (4%) Nasopharyngeal = 7 (8%) Oral cavity = 1 (1%) Oropharyngeal = 44 (53%) Hypopharyngeal = 6 (7%) Laryngeal = 17 (21%) Unknown = 5 (6%)	Tx-2 = 28 (34%) T3-T4 = 55 (66%)	D	Videofluoroscopy PEG dependency	Group 1: CRT (*n* = 70) Group 2: RT (*n* = 13)	12 months	Mean dose to the larynx greater than 41 Gy and a receiving volume greater than 24% showed association with increased PEG dependency and aspiration

Christianen et al. 2015 [50]	*N* = 238	Nasopharyngeal = 8 (3%) Oral cavity = 11 (5%) Oropharyngeal = 71 (30%) Hypopharyngeal = 12 (5%) Laryngeal = 136 (57%)	T1-T2 = 161 (68%) T3-T4 = 77 (32%)	D	Grade of swallowing dysfunction according to the RTOG/EORTC Late Radiation Morbidity Scoring Criteria	Group 1: conventional RT (*n* = 33) Group 2: accelerated RT (*n* = 155) Group 3: CRT (*n* = 50)	6, 12, 18, 24 months	Patterns of swallowing dysfunction may be caused by radiobiological mechanisms of radiation induced damage and recovery. No group differences were found

Cohen et al. 2006 [51]	*N* = 53	Oral cavity = 14 (26%) Oropharyngeal = 11 (21%) Hypopharyngeal = 3 (6%) Laryngeal = 9 (17%) Supraglottic = 13 (24%) Unknown = 3 (6%)	T0 = 3 (6%) T1 = 3 (6%) T2 = 17 (32%) T3 = 30 (56%)	D QoL	PSS-HN Head and Neck RT Questionnaire (selected questions) FACT-H&N	CRT	3, 6, 12, 18, 24, 36, 48, 60 months	Most patients returned to pretreatment function (QoL and performance) by 12 months

Dornfeld et al. 2007 [29]	*N* = 27	Oral cavity = 1 (4%) Oropharyngeal = 16 (59%) Hypopharyngeal = 1 (4%) Laryngeal = 6 (22%) Unknown = 3 (11%)	Tx = 2 (7%) T1 = 6 (22%) T2 = 7 (26%) T3 = 5 (19%) T4 = 7 (26%)	V D QoL	Weight Type of diet Type of speech Presence of PEG tube HNCI	CRT	1 year	Speech, diet, and QoL outcomes showed an inverse relationship with the delivered radiation dose to the larynx

Dijkstra et al. 2007 [52]	*N* = 29	Parotid = 4 (14%) Maxilla = 4 (14%) Gingiva = 2 (7%) Floor of mouth = 3 (10%) Trigonum retromolare = 4 (14%) Oropharyngeal = 8 (27%) Other localization = 4 (14%)	No details provided	D	MIO	RT	12–48 weeks	Increase in mouth opening was significantly less in the group of patients with trismus related to head and neck cancer and is difficult to treat with exercise therapy

Feng et al. 2007 [53]	*N* = 36	Base of tongue = 19 (53%) Tonsil = 12 (33%) Nasopharyngeal = 5 (14%)	T1 = 2 (5%) T2 = 11 (31%) T3 = 9 (25%) T4 = 14 (39%)	D QoL	Videofluoroscopy Esophagogram HNQoL UWQoL EORTC Late Radiation Morbidity Scale	CRT	3 months	Statistically significant dose-volume effect relationships for dysphagia and aspiration were found. Reducing the doses to the swallowing structures may improve swallowing

Feng et al. 2010 [54]	*N* = 73	Base of tongue = 38 (52%) Tonsil = 35 (48%)	T1 = 9 (12%) T2 = 29 (40%) T3 = 17 (23%) T4 = 18 (25%)	D	Videofluoroscopy UWQoL (swallowing question) HNQoL (eating domain) Observer rated dysphagia	CRT	3, 6, 12, 18, 24 months	Long-term measures of swallowing were slightly worse than pretherapy measures

Frowen et al. 2010 [16]	*N* = 81	Base of tongue = 19 (24%) Soft palate = 2 (2%) Tonsil = 26 (32%) Supraglottic = 8 (10%) Hypopharyngeal = 8 (10%) Laryngeal = 18 (22%)	T1 = 11 (14%) T2 = 27 (33%) T3 = 31 (38%) T4 = 12 (15%)	D	Videofluoroscopy	Group 1: CRT (*n* = 23) Group 2: CT (*n* = 58)	3, 6 months	Swallowing in both groups was best at baseline; a decline at 3 months and an improvement at 6 months after therapy were shown. Baseline levels were not reached. Predictors for swallowing outcome were intoxications, tumor size, RT technique, and baseline level of swallowing. Patients who received conformal RT had a very low risk of penetration and aspiration of liquids by 6 months after treatment

Haderlein et al. 2014 [55]	*N* = 45	Oropharyngeal = 3 (7%) Hypopharyngeal = 18 (40%) Laryngeal = 24 (53%)	T2 = 15 (33%) T3 = 17 (38%) T4 = 13 (29%)	D QoL	PEG dependency EORTC QLQ-C30	CRT	3–6- month intervals	Almost 50% of patients had deterioration of swallowing function after CRT

Hutcheson et al. 2014 [56]	*N* = 47	Nasopharyngeal = 1 (2%) Oral cavity = 1 (2%) Oropharyngeal = 41 (88%) Hypopharyngeal = 2 (4%) Supraglottic = 2 (4%)	T1 = 16 (34%) T2 = 14 (30%) T3 = 12 (25%) T4 = 5 (11%)	D	Videofluoroscopy PSS-HN MDADI	Group 1: RT (*n* = 23) Group 2: CRT (*n* = 23) Group 3: surgery (*n* = 1)	6, 12, 24 months	Two years after therapy, mild deterioration of swallowing without chronic aspiration was found

Jacobi et al. 2016 [30]	*N* = 34	Nasopharyngeal = 6 (18%) Oral cavity/oropharyngeal = 15 (44%) Hypopharyngeal = 13 (38%)	T1 = 6 (18%) T2 = 13 (38%) T3 = 11 (32%) T4 = 4 (12%)	V	Speech analysis	CRT	10 weeks; 1 year	Received dose to tongue and velopharynx were most relevant for speech and voice quality

Karlsson et al. 2015 [31]	*N* = 74	Laryngeal = 74 (100%)	T0 = 1 (1%) T1 = 44 (60%) T2 = 22 (30%) T3 = 6 (8%) T4 = 1 (1%)	V QoL	EORTC QLQ-C30 EORTC QLQ-H&N35 S-SECEL	Group 1: CRT + voice rehabilitation (*n* = 37) Group 2: CRT only (*n* = 37)	1, 6 months	Patients treated with voice rehabilitation experienced benefits of therapy on communication and HRQoL

Karlsson et al. 2016 [32]	*N* = 40	Laryngeal = 40 (100%)	Tis = 2 (5%) T1 = 20 (50%) T2 = 13 (33%) T3 = 5 (12%)	V QoL	EORTC QLQ-C30 EORTC QLQ-H&N35 S-SECEL Perceptual and acoustic voice analysis	RT (1 subject received concomitant chemotherapy)	1, 6, 12 months	One year after treatment most outcomes showed no significant improvements compared to baseline measurements

Kazi et al. 2008 [33]	*N* = 21	Hypopharyngeal = 8 (38%) Laryngeal = 10 (48%) Supraglottic = 3 (14%)	Stage III Stage IV No details provided	V	Voice analysis Electroglottography	CRT	1, 6, 12 months	Patients treated with CRT had a better voice quality compared to patients after total laryngectomy

Kerr et al. 2015 [34]	*N* = 200	Tongue base = 77 (38%) Tonsil/soft palate = 123 (62%)	T0-T1 = 42 (21%) T2 = 72 (36%) T3 = 48 (24%) T4 = 38 (19%)	V D	KPS ECOG toxicity and response criteria scale PSS-HN RBHOMS VHI-10 Edmonton Self-Assessment Scale (Self-rated Xerostomia)	Group 1: 3DCRT (*n* = 83) Group 2: IMRT (*n* = 117)	3, 6, 12, 24 months	IMRT showed better functional outcomes compared to 3DCRT, both 3–6 and 12–24 months after treatment

Kotz et al. 2012 [57]	*N* = 26	Nasopharyngeal = 1 (4%) Tongue base = 11 (42%) Tonsil = 11 (42%) Oropharyngeal = 1 (4%) Glottic = 1 (4%) Unknown = 1 (4%)	T2 = 1 (4%) T3 = 5 (19%) T4 = 20 (77%)	D	PSS-HN (eating in public and normalcy of diet) FOIS	Group 1: CRT + prophylactic swallowing therapy (*n* = 13) Group 2: CRT (*n* = 13)	3, 6, 9, 12 months	Prophylactic swallowing therapy improves swallowing at 3 and 6 months; later there were no group differences

Kraaijenga et al. 2014 [35]	*N* = 22	Nasopharyngeal = 4 (18%) Oral cavity/ oropharyngeal = 10 (46%) Hypopharyngeal/ laryngeal = 8 (36%)	T1 = 5 (23%) T2 = 9 (41%) T3 = 7 (32%) T4 = 1 (4%)	V D QoL	Videofluoroscopy Acoustic analysis Presence of feeding tube FOIS Pain (VAS) Trismus QoL aspects (based on EORTC QLQ-C30 and EORTC QLQ-H&N35) SWAL-QoL VHI	CRT	2, 6 years	Functional swallowing and voice problems at 6 years after treatment were minimal, possibly due to preventive swallowing rehabilitation programs

Kumar et al. 2014 [58]	*N* = 46	Tonsil = 19 (41%) Base of tongue = 22 (47%) Pharyngeal wall = 3 (7%) Unknown = 2 (5%)	T0 = 2 (4%) T1 = 15 (33%) T2 = 14 (30%) T3 = 12 (26%) T4 = 3 (7%)	D	Videofluoroscopy	CRT	From <6 to >18 months	Aspiration and penetration were associated with dose and volume delivered to the floor of mouth muscles

Lazarus et al. 2014 [36]	*N* = 29	Nasopharyngeal = 3 (10%) Oropharyngeal = 18 (63%) Pharyngeal = 1 (3%) Hypopharyngeal = 1 (3%) Laryngeal = 5 (18%) Unknown primary = 1 (3%)	Stage I = 2 (7%) Stage II = 1 (4%) Stage III = 5 (17%) Stage IVa = 21 (72%)	D V QoL	Tongue strength, jaw ROM, and tongue ROM Saliva weight Eating Assessment Tool MDADI Speech Handicap Index EORTC QLQ-H&N35 PSS-HN (normalcy of diet, eating in public, and understandability of speech) KPS	CRT	3, 6 months	Patients performed worse in oral outcomes, performance status, and QoL after treatment

List et al. 1999 [59]	*N* = 64	Nasopharyngeal = 1 (2%) Oral cavity = 6 (9%) Oropharyngeal = 34 (53%) Hypopharyngeal = 10 (16%) Laryngeal = 9 (14%) Other = 4 (6%)	Stage III = 4 (6%) Stage IV = 60 (94%)	D QoL	KPS PSS-HN McMaster University Head and Neck RT Questionnaire (selected questions) FACT-H&N	CRT	1, 3, 6, 9, 12 months	Decline of QoL and functional aspects resolved 1 year after treatment; however, oral intake stayed restricted

McLaughlin et al. 2010 [60]	*N* = 91	Nasopharyngeal = 9 (10%) Oral cavity = 19 (21%) Oropharyngeal = 32 (35%) Hypopharyngeal = 7 (8%) Laryngeal = 12 (13%) Unknown = 4 (4%) Other = 8 (9%)	Stage II = 1 (1%) Stage III = 21 (23%) Stage IV = 69 (76%)	D	Weight loss Aspiration Overall nutritional status Duration G-tube placement Treatment-related complications	CRT	6, 12 months	Patients treated with CRT could be managed without nutritional support via G-tube. Dysphagia at baseline and advanced tumor stage are associated with increased risk of longer G-tube dependency

Mittal et al. 2001 [37]	*N* = 39	Nasopharyngeal = 4 (10%) Oropharyngeal = 17 (44%) Hypopharyngeal = 7 (18%) Laryngeal = 5 (13%) Unknown = 6 (15%)	Stage III = 5 (13%) Stage IV = 34 (87%)	D V	Videofluoroscopy Saliva production PSS-HN FACT-H&N Fisher-Logemann Test of Articulation Competence	Group 1: CRT with TDC (*n* = 18) Group 2: CRT without TDC (*n* = 21)	3 months	Patients treated with TDC had better oral intake, swallowing function, and articulation

Murry et al. 1998 [61]	*N* = 37	Oropharyngeal = 19 (52%) Hypopharyngeal = 6 (16%) Laryngeal = 12 (32%)	T3 T4 (No details provided)	D	HNRQ Questionnaire on swallowing	CRT	6 months	During treatment QoL and swallowing function decreased acutely and significantly. Six months after therapy QoL exceeded pretreatment level. Recovery was site-specific: oropharyngeal tumor patients had poorest outcome, whereas hypopharyngeal tumor patients showed most rapid recovery. Physical recovery followed psychosocial recovery. Organ preservation treatment may improve swallowing after treatment

Niedzielska et al. 2010 [38]	*N* = 45	Laryngeal = 45 (100%)	T1 = 24 (53%) T2 = 21 (47%)	V	Videolaryngostroboscopy Acoustic analysis	RT	1–3 years	All irradiated patients showed reduced vibration of the vocal cords. Except for some of the acoustic parameters, most data were comparable to a healthy control group (*N* = 24)

Nourissat et al. 2010 [62]	*N* = 535	Oral cavity = 63 (12%) Oropharyngeal = 17 (3%) Hypopharyngeal = 8 (1%) Supraglottic = 100 (19%) Glottic = 347 (65%)	T1 = 329 (61%) T2 = 206 (39%)	D QoL	Weight KPS EORTC QLQ-C30 Structured general questionnaire	RT	Direct posttherapy	The occurrence of adverse effects of RT appeared to be one of the main reasons for weight loss. Correlations were found between genetic factors associated with the adverse effects of cancer treatments

Ottoson et al. 2014 [63]	*N* = 101	Oral cavity = 20 (20%) Oropharyngeal = 62 (61%) Hypopharyngeal = 8 (8%) Laryngeal = 11 (11%)	T1 = 11 (10%) T2 = 16 (16%) T3 = 28 (28%) T4 = 46 (46%)	D	Videofluoroscopy BMI	RT	5 years	Dysphagia with aspiration was related to unintentional weight loss and a lower BMI

Pauli et al. 2013 [64]	*N* = 75	Sinus, nose = 6 (8%) Salivary gland = 10 (13%) Gingiva, buccal = 6 (8%) Tongue, floor of mouth = 15 (20%) Tonsil = 24 (32%) Base of tongue, oropharyngeal = 11 (15%) Other = 3 (4%)	T0 = 5 (7%) T1 = 13 (17%) T2 = 29 (39%) T3 = 9 (12%) T4 = 18 (24%) Unknown = 1 (1%)	D QoL	MIO Patient-reported outcome Gothenburg Trismus Questionnaire EORTC QLQ-C30 EORTC QLQ-H&N35 HADS	Group 1: surgery (*n* = 8) Group 2: surgery + RT (*n* = 15) Group 3: surgery + CRT (*n* = 6) Group 4: RT (*n* = 16) Group 5: CRT (*n* = 29) Group 6: no treatment (*n* = 1)	3, 6, 12 months	Trismus was a major side effect of the treatment of head and neck cancer and deteriorates HRQoL

Pauloski et al. 2006 [65]	*N* = 170	Nasopharyngeal = 8 (5%) Oral cavity = 15 (9%) Oropharyngeal = 80 (47%) Hypopharyngeal = 14 (8%) Laryngeal = 42 (25%) Unknown = 11 (6%)	Stage IV = 122 (72%) Other = 48 (28%)	D	Videofluoroscopy Oral intake	Group 1: CRT (*n* = 147) Group 2: RT (*n* = 22) Unknown (*n* = 1)	1, 3, 6, 12 months	In both groups limitations in oral intake and diet after cancer treatment were significantly related to reduced laryngeal elevation and reduced cricopharyngeal opening due to treatment

Rademaker et al. 2003 [66]	*N* = 255	Nasopharyngeal = 13 (5%) Oral cavity = 25 (10%) Oropharyngeal = 118 (46%) Hypopharyngeal = 22 (9%) Laryngeal = 59 (23%) Unknown = 18 (7%)	Stage II = 16 (6%) Stage III = 48 (19%) Stage IV = 187 (73%) Unknown = 4 (2%)	D	Percentage of oral intake Food consistencies	CRT	1, 3, 6, 12 months	Eating ability decreased during treatment and improved 12 months after treatment to near pretreatment levels

Remmelts et al. 2013 [39]	*N* = 248	Glottic = 248 (100%)	Tis = 26 (10%) T1a = 103 (42%) T1b = 42 (17%) T2 = 77 (31%)	V	VHI (physical subscale) 5-item questionnaire	Group 1: RT (*n* = 159) Group 2: laser surgery (*n* = 89)	12 months	VHI scores were comparable for both groups. Regarding laryngeal preservation surgery is the treatment of first choice

Salama et al. 2008 [67]	*N* = 95	Nasopharyngeal = 4 (4%) Oral cavity = 8 (9%) Oropharyngeal = 49 (52%) Hypopharyngeal = 5 (5%) Laryngeal = 22 (23%) Other = 7 (7%)	Tx = 7 (7%) T1 = 15 (16%) T2 = 19 (20%) T3 = 16 (17%) T4 = 38 (40%)	D	Videofluoroscopy SPS	CRT	1-2 months	Improvement of swallowing ability compared to baseline was associated with advanced tumor stage

Sanguineti et al. 2014 [40]	*N* = 124	Base of tongue = 54 (43%) Soft palate = 2 (2%) Tonsil = 59 (48%) Pharyngeal wall = 1 (1%) Unknown = 8 (6%)	T0 = 8 (6%) T1 = 37 (30%) T2 = 49 (40%) T3 = 14 (11%) T4 = 16 (13%)	V	CTCAE FACT-HN (items HN4 and HN10)	Group 1: CRT (*n* = 108) Group 2: RT (*n* = 16)	3, 6, 12, 18, 24, 36, 48, 60 months	Mild voice changes were common and strictly correlated to mean dose to larynx and should be kept under 50 Gy

Scrimger et al. 2007 [68]	*N* = 47	Nasopharyngeal = 10 (21%) Oral cavity = 20 (43%) Oropharyngeal = 9 (19%) Hypopharyngeal/ laryngeal = 6 (13%) Unknown primary = 2 (4%)	T0 = 2 (4%) T1 = 7 (15%) T2 = 20 (42%) T3 = 12 (26%) T4 = 6 (13%)	D QoL	Mouth saliva flow UW-QoL RTOG late-toxicity scale XQoL	Group 1: RT (*n* = 5) Group 2: CRT (*n* = 12) Group 3: surgery + RT (*n* = 30)	3, 6, 12 months	Nonsurgery resulted in better QoL questionnaire scores compared to surgery. Patients with good saliva production did not exhibit better QoL after RT than patients with less saliva production

Spector et al. 1999 [41]	*N* = 659	Glottic = 659 (100%)	T1 = 659 (100%)	V	Voice preservation	Group 1: low-dose RT (*n* = 90) Group 2: high-dose RT (*n* = 104) Group 3: conservation surgery (*n* = 404) Group 4: endoscopic resection (*n* = 61)	5 years	Groups 2–4 had similar unaided laryngeal voice preservation rates; however group 1 had significant lower unaided laryngeal voice preservation

Starmer et al. 2014 [69]	*N* = 71	Oropharyngeal = 71 (100%)	T1 = 24 (34%) T2 = 19 (27%) T3 = 13 (18%) T4 = 12 (17%) Unknown = 3 (4%)	D	Videofluoroscopy FOIS	Group 1: CRT (*n* = 65) Group 2: RT (*n* = 6)	1–18 months	Patients undergoing nonsurgical treatment for oropharyngeal tumors were at risk for posttreatment dysphagia

Stenson et al. 2010 [70]	*N* = 111	Buccal = 4 (3%) Alveolus/gingivae = 7 (6%) Floor of mouth = 32 (29%) Tongue = 50 (45%) Palate/oral cavity NOS = 4 (4%) Trigonum retromolare = 13 (12%) Unknown = 1 (1%)	T1 = 9 (8%) T2 = 15 (14%) T3 = 20 (18%) T4 = 67 (60%)	D	Videofluoroscopy SPS	Group 1: CRT (*n* = 84) Group 2: surgery + CRT (*n* = 27)	2, 4, 6, 8, 10, 12, 16, 20, 24, 30, 36 months	Ninety-two percent of all patients were able to maintain weight via oral route. Both groups showed comparable overall survival. Ninety-two percent of all patients had a sufficient oral intake

Strigari et al. 2010 [71]	*N* = 63	Nasopharyngeal = 44 (70%) Floor of mouth/oral cavity = 2 (3%) Oropharyngeal = 11 (17%) Hypopharyngeal = 4 (7%) Unknown primary = 2 (3%)	T1-T2 = 17 (23%) T3-T4 = 46 (73%)	D	Saliva flow Xerostomia related questionnaires RTOG late-toxicity scale	RT	3, 6, 12, 18, 24 months	The mean score on the xerostomia related questionnaire increased (worsened) after RT and decreased (improved) over time in all patients

Tuomi et al. 2015 [42]	*N* = 67	Supraglottic = 13 (19%) Glottic = 54 (81%)	Tis = 2 (3%) T1 = 41 (61%) T2 = 17 (25%) T3 = 6 (9%) T4 = 1 (2%)	V D QoL	Acoustic analysis EORTC QLQ-C30 EORTC QLQ-H&N35 S-SECEL	RT	1 month	Patients treated for supraglottic tumors experienced more problems in eating and swallowing prior to therapy compared to glottic tumors and demonstrated significant HRQoL reduction after treatment. In contrast, glottic tumors presented with inferior voice quality

Urdaniz et al. 2005 [77]	*N* = 60	Paranasal sinuses = 3 (5%) Nasopharyngeal = 3 (5%) Oral cavity = 2 (3%) Oropharyngeal = 25 (42%) Hypopharyngeal = 9 (15%) Laryngeal = 18 (30%)	T2 = 2 (3%) T3 = 20 (33%) T4 = 38 (64%)	QoL	EORTC QLQ-C30 EORTC QLQ-H&N35	Group 1: hyperfractionated concomitant boost RT + cisplatin (*n* = 30) Group 2: hyperfractionated conventional RT + cisplatin (*n* = 30)	1 month	QoL in both groups was relatively good. QoL improved in the follow-up period

Vainshtein et al. 2015 [72]	*N* = 40	Base of tongue = 18 (45%) Tonsil = 22 (55%)	T1 = 8 (20%) T2 = 20 (50%) T3 = 8 (20%) T4 = 4 (10%)	D QoL	HNQoL UWQoL XQoL	CRT	1, 3, 6, 12, 18, 24 months	At 6.5 years after therapy patients showed a stable or improved HRQoL in most domains comparable with baseline and 2 years after therapy

van der Molen et al. 2011 [73]	*N* = 49	Nasopharyngeal = 7 (14%) Oral cavity/ oropharyngeal = 24 (49%) Hypopharyngeal/ laryngeal = 18 (37%)	T1 = 8 (16%) T2 = 15 (31%) T3 = 19 (39%) T4 = 7 (14%)	D	Videofluoroscopy MIO BMI FOIS VAS pain	Group 1: standard rehabilitation (*n* = 28) Group 2: experimental rehabilitation (*n* = 27)	10 weeks	(Preventive) rehabilitation in head and neck cancer patients was feasible and improved functional outcomes after therapy

van der Molen et al. 2012 [44]	*N* = 55	Nonlaryngeal = 36 (65%) Laryngeal = 19 (35%)	T1 = 8 (15%) T2 = 15 (27%) T3 = 21 (38%) T4 = 11 (20%)	V QoL	Acoustic analysis Study-specific QoL questionnaire	CRT	10 weeks; 1 year	CRT effects 10 weeks after therapy were worse than 1 year after therapy, and both were worse than baseline

van der Molen et al. 2013 [74]	*N* = 55	Nasopharyngeal = 7 (13%) Oral cavity/ oropharyngeal = 29 (53%) Hypopharyngeal/ laryngeal = 19 (34%)	T1 = 8 (15%) T2 = 15 (27%) T3 = 21 (38%) T4 = 11 (20%)	D	Videofluoroscopy MIO Study-specific structured questionnaire	CRT	10 weeks; 1 year	A correlation between doses and structures was found for dysphagia and trismus

Verdonck-de Leeuw et al. 1999 [43]	*N* = 60	Glottic = 60 (100%)	T1 = 60 (100%)	V	Videolaryngostroboscopy Voice quality rating Self-rating of vocal performance and quality	RT	0.5–10 years	Voice and its characteristics improved after treatment but did not reach pretreatment levels in half of the patients

Verdonck-de Leeuw et al. 2014 [79]	*N* = 164	Oral/oropharyngeal = 95 (58%) Hypopharyngeal/ laryngeal = 69 (42%)	No details provided	QoL	EORTC QLQ-C30 EORTC QLQ-H&N35	CRT	6 weeks; 6, 12, 18, 24 months	Significant difference in HRQoL between survivors and nonsurvivors in favor of survivors was found

Vlacich et al. 2014 [75]	*N* = 141	Sinus/nasal cavity = 2 (1%) Nasopharyngeal = 12 (9%) Oral cavity = 5 (4%) Oropharyngeal = 82 (58%) Hypopharyngeal = 6 (4%) Laryngeal = 30 (21%) Unknown = 4 (3%)	Stage III = 42 (30%) Stage IVa = 81 (57%) Stage IVb = 18 (13%)	D	PEG requirement	CRT	12 months	IMRT dose to the inferior constrictor correlated with persistent dysphagia requiring prolonged PEG use

Wilson et al. 2011 [76]	*N* = 167	Nasopharyngeal = 5 (3%) Oropharyngeal = 66 (39%) Hypopharyngeal = 21 (13%) Laryngeal = 63 (38%) Unknown primary = 12 (7%)	T1 = 37 (22%) T2 = 37 (22%) T3 = 37 (22%) T4 = 44 (27%) Unknown = 12 (7%)	D QoL	MDADI UWQoL	Group 1: CRT (*n* = 104) Group 2: RT (*n* = 63)	3, 6, 12 months	HRQoL deteriorated significantly after treatment. Little improvement may be expected 3 to 12 months after treatment

3DCRT: 3D conformal radiotherapy; BMI: body mass index; CRT: chemoradiotherapy; CTCAE: common terminology criteria for adverse events; D: digestive tract; ECOG: Eastern Cooperative Oncology Group; EORTC QLQ-C30: European Organization for Research and Treatment of Cancer Quality of Life Questionnaire; EORTC QLQ-H&N35: European Organization for Research and Treatment of Cancer Quality of Life Questionnaire Module Head and Neck Cancer; FACT-HN: functional assessment of cancer therapy-head and neck; FOIS: functional oral intake scale; GRBAS: grade, roughness, breathiness, asthenia, strain scale; HADS: hospital anxiety and depression scale; HNCI: head and neck cancer inventory; HNQoL: head and neck quality of life; HNRQ: head and neck radiotherapy questionnaire; HRQoL: health-related quality of life; IMRT: intensity-modulated radiation therapy; KPS: Karnofsky performance status scale; LENT/SOMA: late effects normal tissue-subjective, objective, management, analytic scales; MDADI: MD Anderson dysphagia inventory; MIO: maximum incisal opening; NOS: not otherwise specified; PEG: percutaneous endoscopic gastrostomy; PSS-HN: performance status scale for head and neck cancer patients; QoL: quality of life; RBHOMS: Royal Brisbane Hospital outcome measure for swallowing; ROM: range of motion; RT: radiotherapy; RTOG: Radiation Therapy Oncology Group; S-SECEL: Swedish version of the self-evaluation of communication experiences after laryngeal cancer; SPS: swallowing performance status scale; TDC: tissue/dose compensation; UWQoL: University of Washington Quality of Life Questionnaire; V: voice and/or speech; VAS: visual analog scale; VHI: voice handicap index; VHI-10: voice handicap index-10; VR-QoL: voice-related quality of life; XQoL: xerostomia questionnaire.

**Table 4:** Overview of speech pathology interventions aimed at addressing problems in dysphagia, speech, voice, and trismus (*n* = 14).

Reference	Topic	General description of intervention and treatment intensity/duration	Description of specific exercises	Conclusions specific to therapy
Agarwal et al. 2009 [23]	Voice	All patients received counseling and voice therapy by a trained speech pathologist No specific data provided on treatment frequency/intensity	No description of exercises provided	Forty-seven of 50 patients showed compliance to the therapy. No specific conclusions of influence of provided therapy on primary outcomes described

Akst et al. 2004 [46]	Swallowing	Swallowing evaluation and intervention when clinically indicated	No description of exercises provided	No specific conclusions of influence of provided therapy on primary outcomes described

Buchbinder et al. 1993 [48]	Trismus	Six to 10 exercise sessions per day for a 10-week period	Group 1: unassisted exercises: reach maximum MIO and closing, jaw motion to left, right, and protrusively Group 2: unassisted exercises: reach maximum MIO and closing, jaw motion to left, right, and protrusively. Stacked tongue depressors, to mechanically increase MIO (5 × 30 seconds per session) Group 3: unassisted exercises: reach maximum MIO and closing, jaw motion to left, right, and protrusively. Combined with the TheraBite System (5 × 30 seconds per session)	The first four weeks no differences between groups were found. After week 4 minimal improvements in groups 1 and 2 were found and group 3 still improved. The highest increment in MIO was reached in group 3

Dijkstra et al. 2007 [52]	Trismus	Physical therapy for trismus, median of 4 sessions	Physical therapy consisting of (i) Active range of motion (ii) Hold and relax (iii) Manual stretching (iv) Joint distraction Following therapeutic tools are used in described cohort: (i) Rubber plugs (ii) Tong blades (iii) Dynamic bite opener (iv) TheraBite System	MIO increases significantly after physical therapy. History of HNC decreases the effect of physical therapy, compared to other trismus patients

Frowen et al. 2010 [16]	Swallowing	All patients were seen by a speech pathologist as an aspect of regular care	No description of exercises provided	No specific conclusions of influence of provided therapy on primary outcomes described

Hutcheson et al. 2014 [56]	Swallowing	All patients received prophylactic swallowing therapy to avoid nothing by mouth periods during treatment No specific data provided on treatment frequency/intensity	Targeted swallowing exercises	No specific conclusions of influence of provided therapy on primary outcomes described

Karlsson et al. 2015 [31]	Voice	Group 1: voice therapy group received 10 × 30-minute sessions over 10 weeks Group 2: vocal hygiene group: 1 session for vocal hygiene advice	Group 1: voice therapy consisting of relaxation, respiration, posture, and phonation exercises Group 2: vocal hygiene advice	Patients treated with voice therapy experienced greater improvements compared to patients that only received vocal hygiene advice. Group 1 showed a significant better functional communication and HRQoL

Kotz et al. 2012 [57]	Swallowing	Group 1: weekly treatment by speech pathologist and daily 3 × 10 home sessions of exercises. Group 2: swallowing assessment and treatment if necessary after treatment	Group 1: prophylactic swallowing therapy consisting of effortful swallow, tongue base retraction exercises, super supraglottic swallow, and the Mendelssohn maneuver Group 2: control group only receive symptomatic dysphagia treatment	Prophylactic swallowing therapy improves swallowing at 3 and 6 months; later there were no group differences found

Kraaijenga et al. 2014 [35]	Swallowing and voice	Daily practice from the start of the treatment until 1 year after treatment	Two combined groups: TheraBite System and standard logopedic swallowing exercises (the same cohort as van der Molen et al. 2011 [73])	Minimal voice and swallowing difficulties were found 60 months after treatment in patients treated with prophylactic swallowing exercises

Sanguineti et al. 2014 [40]	Voice	75.8% of the patients received speech therapy. No therapy was provided to 30 patients No specific data provided on treatment frequency/intensity	No description of exercises provided	No specific conclusions of influence of provided therapy on primary outcomes described

Starmer et al. 2014 [69]	Swallowing	Patients received prophylactic swallowing and trismus exercises	No description of exercises provided	No specific conclusions of influence of provided therapy on primary outcomes described

van der Molen et al. 2011 [73]	Swallowing	Patients received instructions in advance of their oncological treatment. Three times daily exercises	Group 1: range-of-motion exercises and three strengthening exercises, that is, the effortful swallow, the Masako maneuver, and the super supraglottic swallow. Stretch holding for 10–30 seconds at a point of mild discomfort Group 2: stretch of the mouth using the TheraBite System and a strengthening exercise: swallowing with the tongue elevated to the palate while maintaining mouth opening at 50% of its maximum. Stretch holding for 10–30 seconds at a point of mild discomfort	Similar outcomes in both groups were found. Preventive rehabilitation can improve early posttreatment functional outcomes

van der Molen et al. 2012 [44]	Voice	No specific speech or voice therapy	N/A	N/A

van der Molen et al. 2013 [74]	Swallowing and trismus	Study was aimed at describing dose-effect relationships in two treatment groups described in earlier study. References to other published study where treatment regime is described	Group 1: standard exercises Group 2: experimental exercises	Any possible difference between the two included treatment groups is not described, nor possible influence of the respective treatments

HNC: head and neck cancer; HRQoL: health-related quality of life; MIO: maximum incisal opening; ROM: range of motion.
